# A dual perspective on first-session therapeutic alliance: strong predictor of youth mental health and addiction treatment outcome

**DOI:** 10.1007/s00787-020-01503-w

**Published:** 2020-03-10

**Authors:** Patty van Benthem, Renske Spijkerman, Peter Blanken, Marloes Kleinjan, Robert R. J. M. Vermeiren, Vincent M. Hendriks

**Affiliations:** 1 Parnassia Addiction Research Center (PARC), Brijder Addiction Care, Zoutkeetsingel 40, 2512 HN The Hague, The Netherlands; 2 Department of Child and Adolescent Psychiatry, Curium-LUMC, Leiden University Medical Center, Leiden, The Netherlands; 3 Epidemiology and Research Support, Trimbos Institute: Netherlands Institute of Mental Health and Addiction, Utrecht, The Netherlands; 4 Department of Interdisciplinary Social Science, Youth Studies, Utrecht University, Utrecht, The Netherlands; 5 Youz, Parnassia Group, The Hague, The Netherlands

**Keywords:** Therapeutic alliance, Youth, Substance use disorder, Mental health, Treatment outcome

## Abstract

We investigated the potential role of first-session therapeutic alliance ratings to serve as an early marker of treatment outcome in youth mental health and addiction treatment. The present study is among the first to incorporate both a youths’ and a therapists’ perspective of the therapeutic alliance in order to maximize predictive value of the alliance for treatment outcome. One hundred and twenty-seven adolescents participated in a multi-site prospective naturalistic clinical cohort study, with assessments at baseline and at 4 months post-baseline. Main outcome measure was favorable or unfavorable treatment outcome status at 4-month follow-up. Early therapeutic alliance had a medium and robust association with treatment outcome for youth’ (*b* = 1.29) and therapist’ (*b* = 1.12) perspectives and treatment setting. Based on the two alliance perspectives four subgroups were distinguished. Incorporating the alliance-ratings from both perspectives provided a stronger predictor of treatment outcome than using one perspective. Youth with a strong alliance according to both perspectives had an eightfold odds of favorable treatment outcome compared with youth with a weak alliance according to both perspectives. The association between therapeutic alliance and treatment outcome in youth mental health and addiction treatment may be substantially stronger than earlier assumed when both a youths’ and therapists’ perspective on alliance is considered.

## Introduction

Mental disorders with severe impairment—including substance use disorders (SUD)—affect about one in four to five youths aged 13–18 years in their lifetime [1, 2]. For these young people a range of evidence-based treatments is available, including cognitive behavioral therapy and family-based treatments. However, not all youth who receive mental health treatment benefit from treatment. Weisz et al. [3] found a mean between-groups effect-size of Cohen’s *d* = 0.46 (medium effect) at the end of treatment, in a meta-analysis of 447 randomized controlled trials of psychological youth mental health therapies. Treatment effect-sizes differed substantially across the targeted disorders, but remarkably were not moderated by type of treatment offered in the experimental conditions of the trials, although most robust evidence was found for behavioral treatments. With one exception, no studies of youth with SUD were included in Weisz et al. [3]. However, meta-analyses of youth with SUD showed comparable treatment effectiveness across different treatment types [4, 5]. Thus, a considerable proportion of treatment-seeking adolescents do not benefit from mental health/addiction treatment, no treatment is clearly superior, and it is largely unknown which adolescents benefit most from which type(s) of treatment.

This lack of clear differential effectiveness between theoretically quite diverging treatment approaches has led researchers in the mental health field to broaden their focus to common (“non-specific”) factors that are shared by most psychotherapies. From these, the therapeutic alliance—also referred to as working alliance or helping alliance; both terms reflecting the collaborative aspects of the therapist-patient relationship [6]—association with treatment outcome has probably received most attention.

Concerning the association between therapeutic alliance and clinical outcomes of mental health treatment, three meta-analyses were recently published: one on adults [6] and two on youths [7, 8]. In their meta-analysis of 295 studies of psychotherapy among adults, Flückiger et al. [6] found an overall alliance-outcome effect-size of *r* = 0.28 (medium effect), with similar effect-sizes across treatment approaches and across alliance perspectives (e.g., therapist or patient). Notably, the effect-sizes of the alliance-treatment outcome association differed across disorders, with highest values for personality disorders and lowest values for substance use and eating disorders.

With regard to youth, Murphy and Hutton [8] found a pooled alliance-outcome effect-size of *r* = 0.29 (medium effect) in their meta-analysis of 27 youth psychotherapy studies, and Karver et al. [7] found in their meta-analysis of 28 studies an association of *r* = 0.19 (small to medium effect). Effect moderators of the alliance–outcome association in youth were investigated only by Karver et al. [7] and appeared to be comparable with those found by Flückiger et al. [6] in adults: type of treatment (behavioral vs. non-behavioral) and alliance perspective did not moderate the alliance–outcome association, whereas type of disorder did moderate this association, with particularly low effect-sizes for substance use disorders (*r* = 0.01) and eating disorders (*r* = 0.05). Hence, both the strength of the alliance–outcome association and its clinical moderator— type of targeted disorder—found among youth appears to be similar to that found among adults [6–8].

When measuring therapeutic alliance, time of alliance assessment is an important moderator, with—not surprisingly—weaker associations with outcome when alliance is measured early in the therapeutic process [6]. Nevertheless, measuring alliance as early as possible is clinically relevant to allow its use as an early warning sign of potential patient dissatisfaction, premature treatment termination, and/or unfavorable treatment outcome. In addition, various authors have recommended that, when measuring therapeutic alliance, multiple informants should be involved, because different informants are likely to have different views on the quality of the therapeutic relationship [9, 10]. Furthermore, it has been recommended that the alliance evaluations from these multiple sources should be incorporated to gain more insight into the alliance–treatment outcome association [11]. From the 34 independent studies among youth that were included either in the meta-analyses of Karver et al. [7] and/or Murphy & Hutton [8], only 13 studies involved both youth and therapist as informants of early alliance and agreement between both informants was generally low [12–17] or non-significant [18, 19]. Remarkably, the alliance ratings of both sources were not used in any of these studies to investigate their combined predictive value for treatment outcome. In the present study, we aim to fill this gap and (1) investigate the prognostic importance of first-session therapeutic alliance, as perceived by youth and therapist, for outcome of youth mental health and addiction treatment, and (2) examine whether incorporating first-session evaluations from the perspective of both youth and therapist provides more value for predicting treatment outcome than using the evaluations of one perspective.

## Method

### Design

This study was part of the Professional Alliance with Clients in Treatment (PACT) study—a multi-site prospective naturalistic clinical cohort study among adolescents in outpatient youth mental health care (YMHC) and youth addiction care (YAC). For the present study, we used data collected at the first treatment session and at four months post-baseline. We assessed therapeutic alliance as early as possible—i.e. at the end of the first treatment-session—to minimize possible confound due to the effect of early symptom improvement on the perceived therapeutic alliance. We assessed treatment-outcome four months post-baseline because most symptom improvement occurs in these first months of treatment [20–22]. This study was funded by The Netherlands Organization for Health Research and Development (no. 729101014) and approved by the Medical Ethical Board of the University Medical Center Leiden (P.15.001).

### Participants

**Fig. 1:**
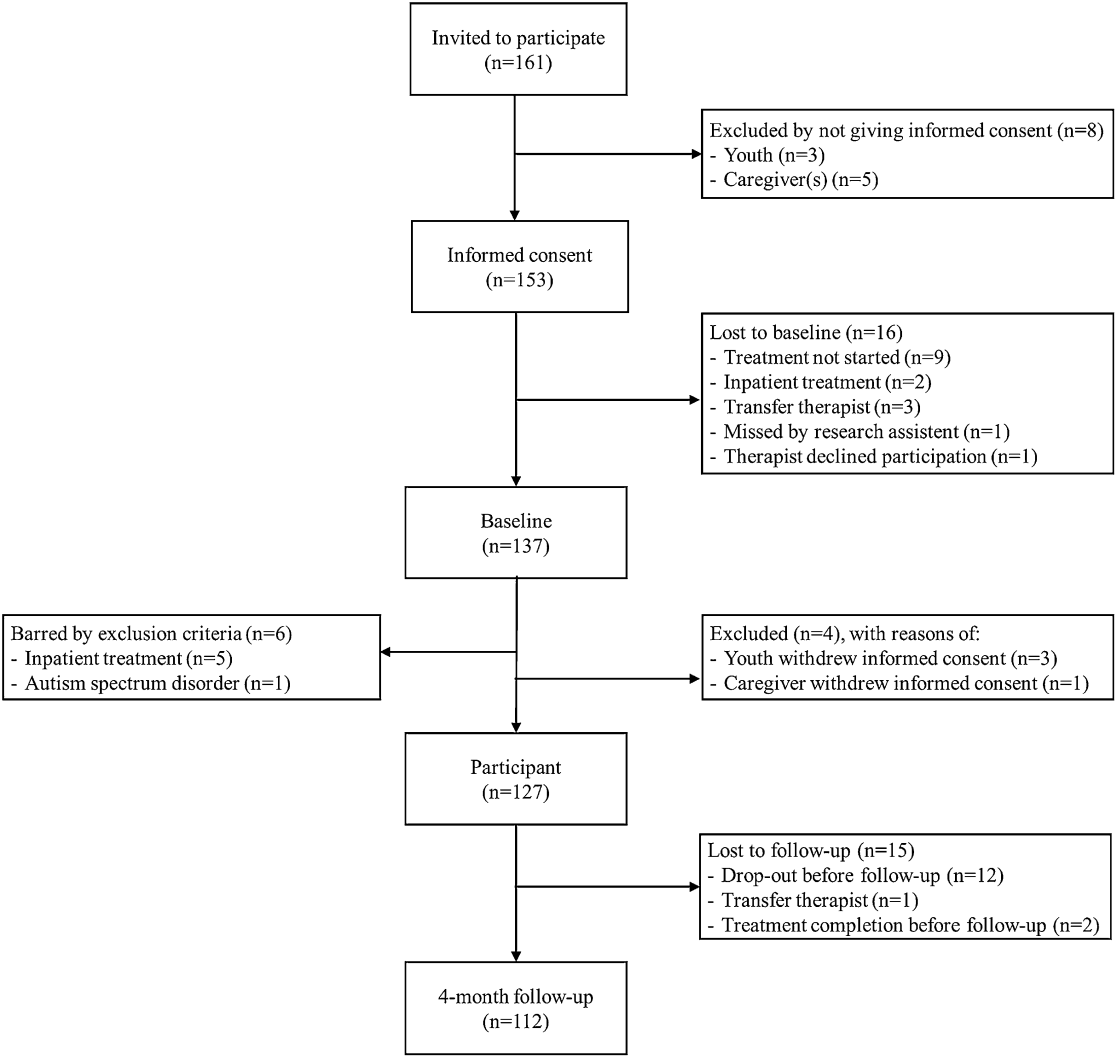
Flowchart of participants

From April 2015 to September 2016, 161 youths were invited to participate in the study from the usual inflow of patients at three YMHC and two YAC facilities in the Netherlands. Eligible patients were 13–23 years old, who started outpatient mental health or addiction treatment, were willing to participate in the study and provided written informed consent (if under the age of 18 years also consent from at least one caregiver). We barred patients from the study if they were cognitively incapable of comprehending the questionnaires (clinical judgement), were diagnosed with DSM-IV autism spectrum disorder, or needed inpatient treatment (clinical judgement). Informed consent was provided by 153 youths and 137 youths (89.5%) completed the baseline assessment (see Fig. 1: Consort Flow diagram). After the baseline assessment, six youths were barred from the study because they needed inpatient treatment (*n* = 5) or were diagnosed with autism spectrum disorder (*n* = 1). Four additional youths were excluded because of withdrawn informed consent. The final sample consisted of 127 youths (YMHC: *N* = 71; YAC: *N* = 56). From these, 15 youths (11.8%) did not participate in the 4-month follow-up assessment (Fig. 1). Fifty-six therapists participated in the study and they treated 1–8 youths each: 23 therapists (41%) treated one youth; 15 (26.8%) treated two youths; and 18 (32.2%) treated three or more youths.

### Treatment

Participants were offered individual outpatient cognitive behavioral interventions (*n* = 93), family-based treatment (*n* = 7) or other treatment (i.e. psychomotor therapy and other psychotherapy, *n* = 24), and type of treatment was not specified for three participants. Median treatment duration was 6 months (interquartile range [IQR]: 4.5–8.0 months) and a median number of 7 sessions was attended (IQR: 4.0–11.0 sessions).

### Assessments

All study assessments, at baseline and 4 months’ follow-up, were conducted by trained research assistants. Study assessments included questions about participants’ and therapists’ demographic background, therapeutic alliance, mental health problems, substance use frequency and diagnosis. Therapeutic alliance was assessed from the perspective of youths and therapists with the Working Alliance Inventory-12 at baseline (WAI-12; [23, 24]; Dutch translation: WAV-12; [25]). The WAI-12 is a 12- item instrument with three subscales: the affective quality of the client-therapist relationship (“Bond”), the degree of agreement on the treatment goals (“Goals”), and the level of task collaboration (“Task”), based on Bordins [26] conceptualization of therapeutic alliance. Youths and therapists were required to rate each item on a 5-point Likert scale ranging from ‘never’ to ‘always’. We used the WAI Total score (range 1–5), as an indication of the overall alliance quality with higher scores indicating better quality of the therapeutic alliance.

To assess youth self-reported mental health problems at baseline and 4 months’ follow-up, the Strengths and Difficulties Questionnaire (SDQ; [27]: Dutch translation: [28]) was administered. The SDQ is a commonly applied screening and treatment-****outcome measure with 25 items with a 3-point Likert scale, ranging from ´not true´ to ´certainly true´. We used the SDQ Total score (range 0–40), with higher scores indicating more problems. The substance use section of the Measurements in the Addictions for Triage and Evaluation, Youth version (MATE-Y; [29]) was used to collect past month information on the youths' primary substance or behavioral addiction (gaming/gambling) at baseline and 4 months follow-up. Clinical psychiatric diagnosis at baseline was made by the treating therapist, who used the criteria of the Diagnostic and Statistical Manual of Mental Disorders [30].

### Primary outcome measure

We used a prespecified dichotomous outcome measure reflecting a favorable versus unfavorable treatment outcome status at 4-month follow-up as the primary outcome measure for both youths in YMHC and YAC. Youths in YMHC were considered to have a favorable outcome status if their 4-month SDQ total score was lower than 12.5. In the absence of formal Dutch cut-off scores [31], we followed the procedures suggested by Jacobson and Truax [32] and De Beurs et al. [33] to determine the cut-off value of 12.5 as the average of the mean SDQ total score in a Dutch general youth population sample (M = 9.7, SD = 4.7; [28]) and the mean baseline SDQ total score in our clinical population (M = 15.3, SD = 5.4). Youths in YAC were considered to have a favorable outcome status if they had used their primary substance or displayed their primary gaming/gambling on less than five days in the 30 days preceding the 4-month follow-up, as recommended in the guidelines for routine outcome monitoring (ROM) in Dutch addiction care (Blanken, et al. 2011, Note from Dutch Expertgroup ROM-Addiction care).

### Data-analysis

Since our alliance data were nested within two levels of clustering we explored the option of using multi-level modeling, but due to insufficient sample size at both levels (level-1: 56 therapists; *M* = 2.27 youth per therapist; range 1–8 and level-2: five treatment facilities, range 4–11 therapists per treatment facility) it was not possible to estimate effects accurately [34]. In order to address the first study goal, pertaining to the prognostic importance of youth- and therapist-rated alliance for treatment outcome, we conducted two separate multivariate logistic regression analyses including either the youth- or the therapist-rated alliance as independent variable and treatment outcome status (favorable versus unfavorable) as dependent variable. In both regression analyses, we examined the effects of potential confounders: gender, age group (≤ 16 or ≥ 17 years), treatment setting (YMHC or YAC), cultural background (Dutch or Non-Dutch), education level (low or high), baseline problem status (favorable or unfavorable) on the primary problem domain (mental health status for youth in YMHC; substance use status for youth in YAC), and baseline problem status (favorable or unfavorable) on the concurrent problem domain (substance use status for youth in YMHC; mental health status for youth in YAC). A variable was considered to be a relevant confounder when the youth- or therapist-rated therapeutic alliance regression coefficient changed with 10% or more after adding the potential confounder into the logistic regression model [35].

In order to address our second study goal, pertaining to the added predictive value for treatment outcome of incorporating the alliance ratings from youths as well as therapists, we conducted a logistic regression analysis in which both youth- and therapist-rated alliance were entered into the model, again with a favorable or unfavorable treatment outcome as dependent variable. A test of improved prediction accuracy was conducted by comparing the difference in -2 log likelihood between the two models, in which we considered a change of ≥ 3.84 (df = 1; *p* = 0.05) as an indication of an improved prediction model.

Finally, we used the median value of the youth-rated and the therapist-rated alliance to distinguish four mutually exclusive subgroups, with alliance rated as (1) “strong” by both youth and therapist, (2) “weak” by the youth and “strong” by the therapist, (3) “strong” by the youth and “weak” by the therapist, and (4) “weak” by both youth and therapist. We then conducted a logistic regression analysis with the subgroup categories as independent variable to predict treatment outcome status, using the first (strong-strong) and the fourth (weak-weak) category as reference category in two separate analyses. To estimate the treatment outcome status of youth with a missing 4-month follow-up assessment, we did not use statistical imputation, but instead asked the treating therapist to provide a 'best estimate' of the youth's outcome status. All statistical analyses were conducted with IBM SPSS Statistics for Windows, version 25.0 (IBM Corp., Armonk, N.Y., USA).

## Results

The baseline characteristics of the study population are summarized in Table 1. Study participants in YMHC (55.9%) were mainly females (74.6%), on average 16.8 years old, and diagnosed with a primary mood (29.6%), anxiety (26.8%) or behavioral (22.5%) disorder. Youth in YAC (44.1%) were predominantly males (83.9%), on average 19.5 years old, and diagnosed with a primary cannabis use disorder (51.8%). Compared with youths in YMHC, youths in YAC were older (*t* (125) =  − 6.95, *p* < 0.001), more often male ($X^{2} \left( 1 \right)$  = 42.99, *p* < 0.001) and more often had an unfavorable problem status on the concurrent problem domain ($X^{2} \left( 1 \right)$ = 16.80, *p* < 0.001).

**Table 1:** Participant characteristics at baseline

	Youth mental health care (*n* = 71) %/mean (sd) median	Youth addiction care (*n* = 56) %/mean (sd) median	Total sample (*n* = 127) %/mean (sd) median
Demographic background			
Age (13–23) (years)	16.8 (2.1) **16.0**	19.5 (2.2) **20.0**	18.0 (2.5) **18.0**
Aged ≤ 16 (%)	53.5	16.1	37.0
Male (%)	25.4	83.9	51.2
Cultural background Non-Dutch (%)	25.4	23.2	24.4
Education level low (%)	64.8	58.9	62.2
Substance use			
Primary substance/addictive disorder (%)			
Cannabis use disorder	–	51.8	22.8
Gaming/gambling disorder	–	17.9	7.9
Alcohol use disorder	–	16.1	7.1
Hard drug use disorder	–	14.3	6.3
Days primary substance use/problem behavior past month	5.4 (9.9) **0.0**	14.3 (12.3)** 13.0**	9.3 (11.9) **2.0**
Problematic substance use past month (≥ 5 days) (%)	25.4	62.5	41.7
Mental health			
Primary non-addiction disorder (%)			
Mood disorder	29.6	–	16.5
Anxiety disorder	26.8	–	15.0
Behavioral disorder	22.5	–	12.6
Attention Deficit Hyperactivity Disorder (ADHD)	7.0	–	3.9
Other disorder	14.1	–	7.9
Strengths and Difficulties Questionnaire (SDQ score: 0–40)	15.4 (5.4)** 16.0**	15.1 (5.6)** 15.0**	15.3 (5.4)** 15.0**
Problematic mental health status (SDQ score ≥ 12.5) (%)	69.0	66.1	67.7
Treatment			
Treatment type (%)			
Cognitive behavioral interventions	58.0	96.4	75.0
Family-based treatment	7.2	3.6	5.6
Other	34.8	–	19.4
Concurrent pharmacological treatment: Yes (%)	26.5	7.3	17.9
Therapeutic alliance			
Youth-rated WAI (1–5)	3.8 (0.7)** 3.9**	4.0 (0.6)** 4.1**	3.9 (0.7)** 4.1**
Therapist-rated WAI (1–5)	3.8 (0.5)** 4.0**	4.0 (0.5)** 4.1**	3.9 (0.5)** 4.0**

### 4-month treatment outcome

The proportion of youths with an unfavorable problem status decreased from 66.1% at baseline to 56.7% at 4 months’ follow-up (McNemar $X^{2}$ test, *p* = 0.052).

### Baseline therapeutic alliance as predictor for 4-month treatment outcome

The unadjusted regression coefficient of baseline youth-rated alliance for predicting outcome was *b* = 1.18 (*p* < 0.001; OR 3.25) (Table 2). From all potential confounders, only ‘gender’ (highlighted in bold in Table 2) changed the regression coefficient of the association between youth-rated therapeutic alliance and 4-month treatment outcome with more than 10% (from 1.18 to 1.29) and was, therefore, considered to be a relevant confounder. In the final model, the youth-rated therapeutic alliance–outcome association adjusted for gender was *b* = 1.29 (*p* < 0.001; OR 3.65).

**Table 2:** Unadjusted and adjusted associations between therapeutic alliance and 4-month treatment outcome

	Youth-rated therapeutic alliance			Therapist-rated therapeutic alliance		
	b	OR	95%-CI of OR	b	OR	95%-CI of OR
Unadjusted model	1.18	3.25	1.67–6.30	1.10	3.02	1.39–6.57
Adjusted for						
Gender	**1.29**	**3.65**	**1.82–7.29**	0.80	2.67	1.23–5.82
Treatment setting	1.15	3.14	1.62–6.12	1.04	2.83	1.29–6.21
Age group	1.17	3.21	1.65–6.25	**0.99**	**2.70**	**1.25–5.85**
Cultural background	1.19	3.30	1.69–6.45	1.10	3.01	1.38–6.55
Education	1.17	3.24	1.67–6.27	1.12	3.07	1.40–6.71
Concurrent problem domain^a^	1.18	3.27	1.68–6.35	1.09	2.98	1.36–6.52
Baseline problem status	1.12	3.06	1.46–6.41	**1.20**	**3.32**	**1.38–7.98**
Adjusted final model	1.29^b^	3.65	1.82–7.29	1.12^c^	3.07	1.24–7.61

Relevant confounders (≥ 10% change in b) in bold

^a^Substance use status for youth in YMHC; mental health status for youth in YAC

^b^Adjusted for gender

^c^Adjusted for age group and baseline problem status

The unadjusted regression coefficient for therapist-rated alliance was *b* = 1.10 (*p* < 0.005; OR 3.02). Relevant confounders for the association between therapist-rated alliance and 4-month treatment outcome were ‘age group’ and ‘baseline problem status’ (highlighted in bold in Table 2). In the final model, the therapist-rated therapeutic alliance–outcome association adjusted for age group and baseline problem status was *b* = 1.12 (*p *< 0.05; OR 3.07).

### Incorporating both alliance perspectives to predict 4-month treatment outcome

The youths’ and therapists’ alliance ratings were not interrelated (Spearman’s rank correlation = 0.08; *p* = 0.38). Concerning the predictive value for treatment outcome of incorporating the alliance ratings of youths and therapists, we found that adding the second perspective significantly improved the logistic regression model, given a 10.75 (${\rm X}^{2}$ (1, N = 125) = 10.75, *p* = 0.001) change in   −  2 log likelihood. To illustrate the prognostic significance of incorporating both perspectives in clinical practice, we distinguished four subgroups (Table 3). In the subgroup where both youth and therapist considered the first-session alliance as weak (24.8% of the study sample), the proportion of youths with a favorable 4-month outcome amounted to 22.6%, and in the subgroup where both raters considered the alliance as strong (29.6%) this proportion amounted to 70.3%. Compared with the subgroup with strong youth- and strong therapist-rated alliance, the odds ratio of a favorable treatment outcome was significantly lower in each of the three other subgroups (Table 3). Conversely, the odds of a favorable outcome in the subgroup with a strong alliance according to both perspectives were more than eight times higher (large effect) than the odds of a favorable outcome in the subgroup with a weak alliance from both perspectives (OR = 8.10; 95%-CI 2.70–24.30, *p* < 0.001).

**Table 3:** Incorporating alliance ratings of youths and therapists to predict 4-month treatment outcome

	Ratin youth	Rating therapist^a^	*n* (%)	% favorable 4-month treatment outcome	OR	95%-CI	*p*
Therapeutic alliance	Weak	Weak	31 (24.8)	22.6	0.12	0.04–0.37	< .001
	Strong	Weak	25 (20.0)	32.0	0.20	0.07–0.60	< .05
	Weak	Strong	32 (25.6)	40.6	0.29	0.11–0.79	< .05
	Strong^b^	Strong^b^	37 (29.6)	70.3	–	–	–

^a^*n* = 125; two therapist-rated therapeutic alliance missings

^b^Reference group

## Discussion

The aims of our study were to investigate the prognostic importance of first-session youth- and therapist-rated therapeutic alliance and the added predictive value of incorporating these alliance ratings for outcome of youth in YMHC and YAC. Youth- and therapist-rated alliance were not related to each other and each showed a medium association with treatment outcome in both YMHC and YAC. Our findings suggest that incorporating the alliance perspectives of both youths and therapists provides substantially stronger predictive value for treatment outcome than using one perspective only. To illustrate the relevance of this finding for clinical practice, we distinguished four subgroups based on the alliance ratings from both perspectives, and found these subgroups to differ considerably in treatment outcome, with the lowest proportion of favorable outcome in the subgroup where both youth and therapist considered the alliance as weak (23%), and the highest proportion in the subgroup where both raters considered the alliance as strong (70%).

Overall, the medium effect sizes of the alliance–outcome associations in our study are in line with the effect sizes found in previous systematic reviews on therapeutic alliance in adolescents [7, 8] and adults [6]. However, we found similar alliance–outcome associations for SUD as for non-SUD mental health disorders, whereas Fluckiger et al. [6] and Karver et al. [7] reported lower associations for substance use disorders. Our finding that youth- and therapist-rated alliance were not interrelated matches previous research showing nonsignificant correlations between both at the start of treatment [11, 18], and these findings suggest that youth and therapist alliance perspectives differ substantially. Most previous studies that have investigated therapeutic alliance from more than one perspective focused on the issue which of these perspectives showed the strongest association with treatment outcome. To the best of our knowledge, our study is the first in which the alliance evaluations from both the therapist and youth were incorporated to maximize their predictive value for treatment outcome.

Our findings further suggest that it may be worthwhile to start evaluating the therapeutic relationship when initiating therapy. For therapists offering psychosocial services to youth, paying attention to first-session therapeutic alliance may be particularly relevant since youths generally enter treatment on account of parental and environmental concerns and often show distrust of adult authority and have a strong desire for autonomy [36, 37]. Therapists can employ different alliance building techniques to improve therapeutic alliance [38, 39]. Furthermore, regular measurement of the therapeutic alliance throughout the course of treatment may be part of a broader approach, such as Feedback Informed Treatment (FIT) [40] and can be applied in different forms and through different feedback systems [41]. A recent meta-analysis on the application of FIT in psychological services with youth showed an overall beneficial effect in the small range [42]. Although promising, more research on the application of FIT in youth mental health care is warranted.

Our study has several limitations that should be mentioned. First, the use of multi-level modeling was precluded because of the majority of therapists with few (< 2) participating youths; the minority of therapists with a higher number (3–8) of participating youths; and the limited therapists per treatment facility. Nevertheless, we found no difference in alliance ratings between therapists with few participating youths versus therapists with more participating youths; in addition, we found that treatment setting was not a relevant confounder in this study. Second, our findings regarding the association between therapeutic alliance and treatment outcome are merely correlational in nature. To allow stronger causal inferences, Cuijpers and colleagues [43] argue that there should be a temporal association, a dose–response relationship, exclusion of alternative potential mediators, preferably experimental manipulation, and a plausible theoretical explanatory framework. From these, we did take the temporal association into account, as well as possible confounders and moderators of the association, and we measured the alliance as early as possible in the therapeutic process to minimize potential confound due to early symptom improvement. In addition, we believe that the theoretical framework pertaining to the role of common factors like alliance in therapy is not weaker than that of, e.g., specific factors in cognitive behavioral therapy. Third, the focus on month-4 treatment outcome and the lack of long-term follow-up data does not allow for conclusions about the relevance of initial alliance ratings for predicting long-term outcome. Nevertheless, there is evidence that most symptom improvement occurs during the first phase of treatment [20–22, 44].

To conclude, this study supports the importance of including both perspectives on therapeutic alliance for treatment outcome in youth and suggests that incorporating both youths’ and therapists’ alliance perspectives has more prognostic value than considering only one perspective.
